# Beneficial mutualistic fungus *Suillus luteus* provided excellent buffering insurance in Scots pine defense responses under pathogen challenge at transcriptome level

**DOI:** 10.1186/s12870-024-06026-z

**Published:** 2025-01-03

**Authors:** Zilan Wen, Minna J. Manninen, Fred O. Asiegbu

**Affiliations:** Forest Pathology Research Lab, Faculty of Agriculture and Forestry, Department of Forest Sciences, University of Helsinki, Helsinki, 00790 Finland

**Keywords:** Beneficial and mutualistic fungi, Co-inoculation, Plant defense response, Plant growth, *Suillus*, *Heterobasidion*

## Abstract

**Background:**

Mutualistic mycorrhiza fungi that live in symbiosis with plants facilitates nutrient and water acquisition, improving tree growth and performance. In this study, we evaluated the potential of mutualistic fungal inoculation to improve the growth and disease resistance of Scots pine (*Pinus sylvestris* L.) against the forest pathogen *Heterobasidion annosum*.

**Results:**

In co-inoculation experiment, Scots pine seedlings were pre-inoculated with mutualistic beneficial fungus (*Suillus luteus*) prior to *H. annosum* infection. The result revealed that inoculation with beneficial fungus promoted plant root growth. Transcriptome analyses revealed that co-inoculated plants and plants inoculated with beneficial fungus shared some similarities in defense gene responses. However, pathogen infection alone had unique sets of genes encoding pathogenesis-related (PR) proteins, phenylpropanoid pathway/lignin biosynthesis, flavonoid biosynthesis, chalcone/stilbene biosynthesis, ethylene signaling pathway, JA signaling pathway, cell remodeling and growth, transporters, and fungal recognition. On the other hand, beneficial fungus inoculation repressed the expression of PR proteins, and other defense-related genes such as laccases, chalcone/stilbene synthases, terpene synthases, cytochrome P450s. The co-inoculated plants did not equally enhance the induction of PR genes, chalcone/stilbene biosynthesis, however genes related to cell wall growth, water and nutrient transporters, phenylpropanoid/lignin biosynthesis/flavonoid biosynthesis, and hormone signaling were induced.

**Conclusion:**

*S. luteus* promoted mutualistic interaction by suppressing plant defense responses. Pre-inoculation of Scots pine seedlings with beneficial fungus *S. luteus* prior to pathogen challenge promoted primary root growth, as well as had a balancing buffering role in plant defense responses and cell growth at transcriptome level.

**Supplementary Information:**

The online version contains supplementary material available at 10.1186/s12870-024-06026-z.

## Background

Mutualistic fungi that live in symbiosis with plants play pivotal roles in the recycling of carbon, nitrogen, phosphorus, and other nutrients in the boreal forest ecosystem, many of which belong to ectomycorrhiza group. Ectomycorrhizal (ECM) fungi wrap around host lateral roots to form fungal mantle and form hartig net between epidermal and cortical root cells [1]. Ectomycorrhizal fungi (ECF) that form symbiosis with trees has the capacity to influence growth limiting nutrient resources in forest ecosystem [2]. The authors further noted that EMF composition was associated to a three-fold difference in tree growth and that fast tree growth was linked with EMF that harbored high inorganic nitrogen acquisition genes. ECM fungi have received in recent years heightened attention as key mediators which function within common mycorrhizal networks [3–6], which are associated with water and nutrient mobilization [7], plant growth in symbiotic performance [8–10], tree transcriptome in ectomycorrhizal symbiosis [7, 11–15]. Rudawski et al. [16] noted the dominance of several ECM (*Suillus luteus, Rhizopogon roseolus, Thelephora terrestris, Hebeloma crustuliniforme*) in Scots pine seedlings in forest nursery. Policelli et al. [17] reported that ECM helps temperate and boreal forest trees to tolerate harsh environmental conditions such as restoration of sites degraded due to clearcut logging and wildfire, affected by soil erosion and contaminated with heavy metals as well as restoring sites invaded by non-native plant species.

Unlike the main saprotrophic decomposers which dominate in freshly produced organic matter, ECM fungi dominate in deeper soil layers [18]. They potentially act as decomposers, mobilizing N from the soil organic matter pool and making it available to their host plants [19, 20]. ECM fungi have nutrient transporters in their genome for nutrient mobilization [1, 21]. ECM fungi have a limited capacity to decompose plant litter as revealed by the analysis of genome sequences [22, 23], including *Laccaria bicolor* [24], *Tuber melanosporum* [25] and mycorrhizal *Amanita* species [26]. Genome analysis on those ECM fungi indicated the loss of some genes encoding plant cell wall degrading enzymes such as glycoside hydrolases and peroxidases compared to their saprotrophic ancestors [20, 27]. The reductions and losses in specific protein families could be an indication of adaptation of ectomycorrhizal biotrophy in plant tissues [27].

Host plants are the principal sources of metabolic carbon for ECM fungi, which in turn also benefit from ectomycorrhizal colonization. ECM fungi promote plant growth by enhancing water and nutrient uptake especially nitrogen and phosphorus [21]. Root biomass and length of *Populus nigra* increased under ECM fungus inoculation [28]. Several ECM fungi could change tree root architecture (including overall root growth, primary and lateral root length, lateral root number), in which plant hormones signaling and auxin pathways could be involved [29]. ECM can also improve plant performance by inducing local and systemic defense responses which may be controlled by signaling networks involved in plant hormones salicylic acid (SA), jasmonic acid (JA), and ethylene, to confer broad-spectrum resistance during subsequent plant pathogen or herbivore attack [13, 30].

*Suillus luteus*, which is also called slipper Jack, is a common ECM fungus with pines, such as *Pinus sylvestris* [31, 32], *P. elliottii* [33]. *S. luteus* had the ability to help *P. massoniana* absorb phosphorus under nutrient deficiency [34]. *S. luteus* promoted the growth of *P. massoniana* seedlings, producing phytohormones, especially SA and indole-3-carboxylic acid (ICA), methyl indole-3-acetate (ME-IAA), and indole-3-acetic acid (IAA) in mycelium [33]. Stone pine without *S. luteus* symbionts could be more easily susceptible to *H. annosum* than plants with ectomycorrhizal symbiosis [35]. Mycorrhizal inoculation of *Suillus* spp. increased chlorophyll a and b, carotenoids, and soluble protein in Scots pine young seedlings, and increased the activities of plant antioxidant enzymes CAT and POD, and plant beta-1,3-glucanase [36].

Scots pine, dominating in boreal forest, is highly susceptible to infection by necrotrophic basidiomycete *Heterobasidion annosum* sensu stricto (Fr.) Bref. [37]. Aerial basidiospores of the fungal pathogen fall on freshly cut stumps and form invasive hyphae, followed by spreading to nearby trees via root contact [37, 38]. The fungal pathogen grows necrotrophically to get nutrients from the living tree tissues and then turns to saprotrophic growth in dead wood cells [38], which may lead to reduced volume growth and tree mortality. Tree defense responses in conifer-*Heterobasidion* pathosystem demonstrated that the phenylpropanoid pathway, lignin biosynthesis and polymerization, flavonoids biosynthesis, terpenoid and stilbene pathways, and pathogenesis-related (PR) proteins are the principal responses at the gene level [39–46]. Jasmonic acid and ethylene signaling pathway plays a central role in tree defense responses to *Heterobasidion* infection, without antagonism of salicylate-mediated signaling pathway [47, 48].

Plant defense responses to *Heterobasidion* infection and beneficial microbe inoculation such as endophyte have been recorded [48, 49]. However, there are limited studies on plant response to ECM fungi in the presence of pathogenic fungi. The hypothesis is that pre-inoculation of plants with ECM fungi before fungal pathogen challenge would improve plant performance and help plant defense against pathogen infection. We investigated the similarities and difference in host responses between pathogenic and mutualistic fungal interaction, host responses towards pathogenic attack that were maintained under co-inoculation, and host responses towards pathogenic attack that are dampened by the presence of ECM fungus.

## Materials and methods

### Mutualistic beneficial fungus and plant materials

*Suillus luteus* was obtained from the University of Helsinki Fungal Biotechnology Culture Collection (HAMBI/FBCC). Heterokaryotic *Heterobasidion annosum* 02034 was obtained from the culture collection of Kari Korhonen. Scots pine seeds were kindly provided by Natural Resources Institute of Finland (Luke). *S. luteus* was stocked on Modified Melin-Norkrans medium (MMN: CaCl_2_ 0.05 g, NaCl, 0.025 g, MgSO_4_·7H_2_O 0.15 g, FeEDTA 0.0168 g, Thiamine HCl 0.01 g, malt extract 5 g, NH_4_H_2_PO_4_ 0.25 g, KH_2_PO_4_ 0.5 g, glucose 3.0 g, yeast extract 1.0 g, Distilled water 1000 ml). *H. annosum* was maintained on malt extract agar (MEA: malt extract 20 g, agar 15 g, Distilled water 1000 ml). Scots pine seeds were surface sterilized with 30% H_2_O_2_ for 15 min, rinsed several times with sterile water and stratified for 3–4 days in the dark at 4 ◦C. Rows of seeds were laid on 12*12 cm sterile square Petri dishes with 1% water agar and covered with moist, sterile filter paper. The Petri dishes containing seeds were sealed with parafilm, placed in a growth chamber for germination under a photoperiod of 16 h at 20 ◦C for 2 weeks.

### Dual cultures on artificial agar media

Dual cultures of *H. annosum* and *S. luteus* were set up on MMN media to investigate the impact of the ECM fungus on the pathogenic fungus growth. *S. luteus* agar plug (5-mm diameter) was put on the agar media 13 days before adding *H. annosum* agar plug, as the former grows slower than the latter. The distance between the two plugs was 6 cm on Petri dish. Self-pairing refers to two separate agar plugs of the same fungus on the culture plate and served as the control. The fungal cultures grew at 20°C in the dark.

### Seedling inoculation and sampling

*S. luteus* was cultured on a 9-cm circular Petri dish with MMN agar media. One month later, agar plugs on the edge of the fungal culture with active mycelia were moved to a 12-cm square Petri dish with MMN agar media and cellophane membrane. *S. luteus* was growing on membrane agar plates for 17 days, and then the membrane with the fungal mycelia was moved into a new 12-cm square Petri dish containing MMN agar media.

Two-week-old Scots pine seedlings were placed on the top of the fungal mycelia in the square Petri dish with MMN. Fungal mycelia and plant seedlings were grown together on Petri dish for one month to ensure successful inoculation and establishment of the ECM fungus on the plant. One month later, ECM fungus-inoculated seedlings were transferred into soil. The soil was peat-based substrate provided by Kekkilä Professional (Vantaa, Finland). Dry soil was mixed with water, and the moist soil was autoclaved at 121°C for one hour (two times) with an interval of 48 h. The autoclaved soil was put into the 12 cm-sterile square plates. Three or four infected seedlings were transferred into each soil plate. Seedlings were grown in the soil for one month before adding *H. annosum*. *H. annosum* used for the inoculation was pre-incubated in the malt agar media for 3 weeks. Ten agar plugs (0.5 cm in diameter) with active fungal mycelia were introduced into the soil next to the plant roots. The seedlings were watered every two weeks. In total, there were four groups of seedlings, including control seedlings without any inoculum (Ctr), Mutualistic fungus-inoculated seedlings (Sl), pathogen-infected seedlings (Ha), and co-inoculated seedlings with both the ECM fungus and the pathogen (SlHa, or co-inoculation). There were at least three Petri dishes for each group which served as biological replicates, with 3–4 seedlings in each dish. Seedlings were grown with *H. annosum* together for one month before sampling. At the time of sampling, Scots pine seedlings were three and half months old. By then, *S. luteus* had grown with the plants for three months, while *H. annosum* was with the plants for one month. The number of the lateral roots, the length of primary roots of the seedlings were counted and measured at termination of the experiment.

### RNA extraction, library construction, and differential gene expression analysis

Seedling roots were collected for RNA isolation as previously described [49, 50]. Transcriptome sequencing (paired-end, 101bp) was performed at CeGat (Germany). Sequencing libraries were generated by using TruSeq Stranded mRNA (Illumina). The library preparations were sequenced on Illumina NovaSeq 6000. Raw reads were preprocessed by CeGat. Demultiplexing of the sequencing reads was performed with Illumina bcl2fastq (2.20). Adapters were trimmed with Skewer (version 0.2.2). Trimmed raw reads were aligned to the annotated contigs of *Pinus taeda* v2.01 [51] using the Illumina DRAGEN platform (software version 3.10.4). As *Pinus sylvestris* has presently no well annotated genome, thus we used *P. taeda* as the mapping genome. The *P. taeda* genome was also used for the analysis of *P. sylvestris* transcriptomic data partly because the *P. taeda* and *P. sylvestris* sequences showed high level of sequence similarity [52].

The raw reads count was used to identify differentially expressed genes in Ha, Sl, and SlHa compared to Ctr using edgeR package in R v.4.3.3. Genes with at least 1 count per million in at least 3 libraries were utilized for further analysis. Normalized counts obtained by DESeq2's median ratios were transformed using rlog transformation to improve the distance or clustering for principal component analysis (PCA) and hierarchical clustering visualization. The cutoff values for defining differentially expressed genes (DEGs) were false discovery rate adjusted *P*-value (Benjamini and Hochberg’s approach) = 0.05 and |log2(fold change) |= 1.

### Functional annotation

Coding sequences (CDS) of *P. taeda* were extracted from PlantGenIE. The CDS sequences of genes with at least 1 count per million in at least 3 libraries were blasted to Scots pine de novo transcriptome assembly fasta sequences constructed with Trinity [53]. Since all Trinity assembly transcripts had been also annotated [53], we were able to gain the annotation information with nucleotide blast. The best hits were selected by bit scores. Gene ontology (GO) enrichment of DEGs was analyzed by clusterProfiler [54]. R packages such as ggplots, enrichplot, pheatmap were utilized for dotplots, GO maps, and heatmaps.

## Results

The interaction between the ECM fungus and the pathogen in dual agar culture was found to be neither antagonistic nor antibiosis (Additional file 1).

**Fig. 1:**
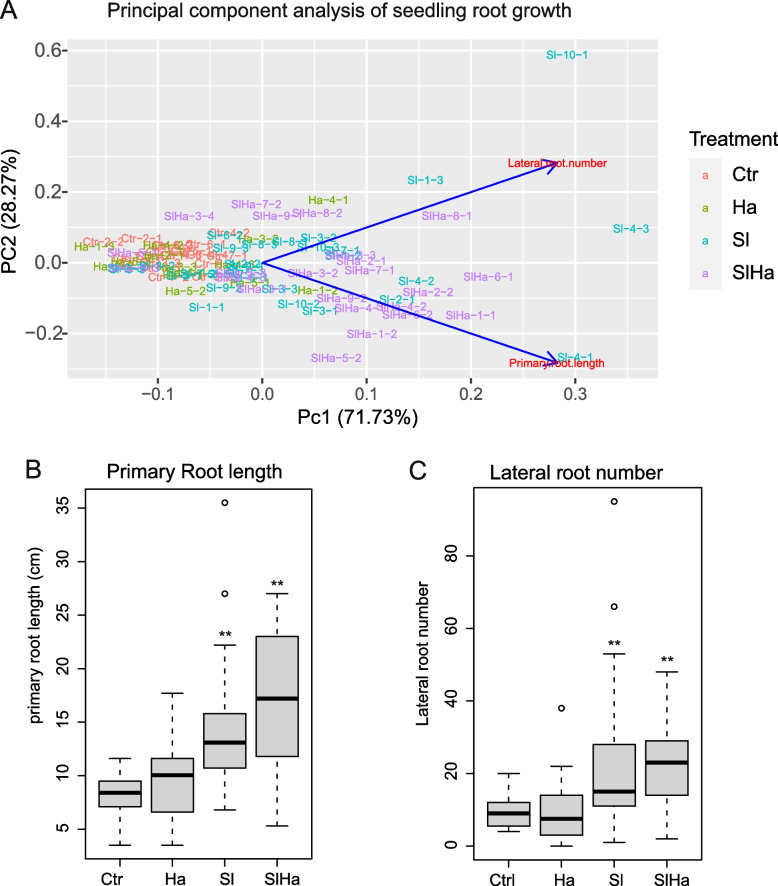
The growth of Scots pine seedlings with fungal infection. **A** Principal component analysis based on the primary root length and lateral root number. **B** Box plot about primary root length of seedlings. **C** Box plot about lateral root number. Ctr: non-inoculated seedlings control; Ha: *Heterobasidion annosum*-inoculated seedlings; Sl: *Suillus luteus*-inoculated seedlings; SlHa: *H. annosum*-infected seedlings in the presence of *S. luteus* (co-inoculated plants). Asterisks indicate whether significant difference exists in the primary root length or lateral root number of treatment such as Ha, Sl, SlHa compared to that of Ctr. (*p* < 0.001: ***; *p* < 0.01: **; *p* < 0.05: *)

**Fig. 2:**
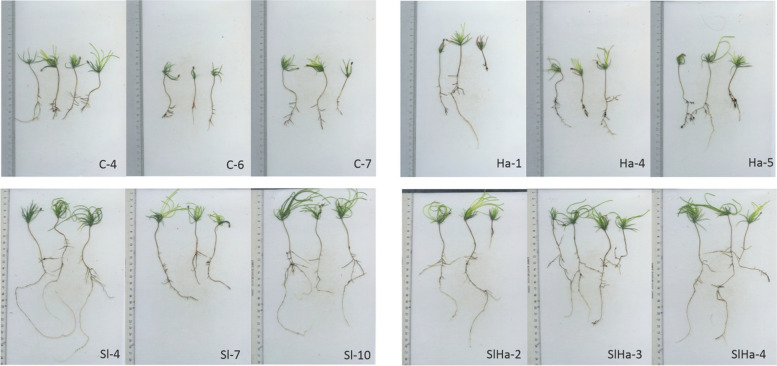
Images of Scots pines seedlings used for RNA sequencing. C: non-inoculated seedlings control; Ha: *H. annosum*-inoculated seedlings; Sl: *Suillus luteus*-inoculated seedlings; SlHa: *H. annosum*-infected seedlings in the presence of *S. luteus* (co-inoculated plants). Numbers refer to the Petri dish ID in Additional file 2

The two fungi were then inoculated either alone or together on roots of Scots pine seedlings. To ensure successful colonization of the roots, *S. luteus* was grown with plant seedlings for a month on artificial media before being transferred to the soil. However, mycorrhization marked by mantle formation or hartig net was not observed. A total of 88 seedlings were harvested (Additional file 2), including 22 seedlings from control (Ctr), 14 seedlings from *H. annosum* infection (Ha), 29 seedlings from *S. luteus* inoculation (Sl), and 23 seedlings from co-inoculation (SlHa) and used for phenotyping. While 38 seedlings were used for RNA sequencing as shown in Fig. 2. Principal component analysis based on the primary root length and lateral root number showed that growth of Ha and Ctr could be different from Sl and SlHa (Fig. 1A). The median values of primary root length (Fig. 1B) in Sl and SlHa were 13.1 cm and 17.2 cm, significantly higher than the values in Ctr and Ha (8.4 cm, 10 cm). The median values of the number of lateral roots in Sl and SlHa were 15 and 23, while the values in Ctr and Ha were 9 and 7.5, respectively (Fig. 1C). We found that seedlings in both Ha and Ctr grew poorly, suggesting pathogenic or other forms of stress in the two conditions. However, the plant root growth was improved significantly with the presence of *S. luteus* (Figs. 1B, 2). Therefore, we reasoned that *S. luteus* as a beneficial fungus promoted plant root growth. Consequently, the term mutualistic or beneficial was utilized to qualify the interaction in this study.

### The RNAseq and transcriptome analysis

**Fig. 3:**
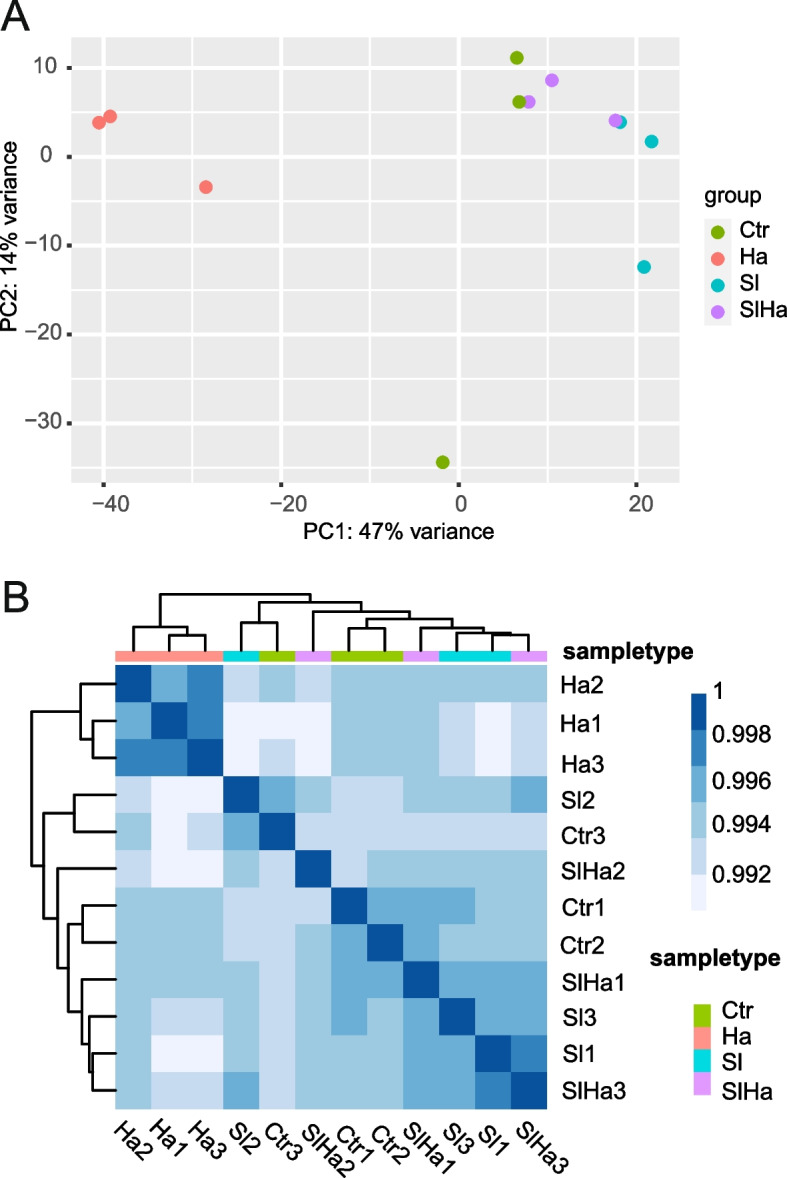
RNAseq profiling of Scots pine in Ctr, Ha, Sl and SlHa. **A** PCA plot of rlog-transformed normalized counts obtained by DESeq2's median of ratios. **B** Heatmap of hierarchical clustering of normalized counts

About 73%—86% of sequenced fragments were mapped to *Pinus taeda* genome, and 35%-40% of the mapping fragments were used for expression quantification (Table 1). The results seem to show that the presence of *S. luteus* appeared to have a balancing buffering effect on the plant stress response in the presence of *H. annosum*. This is partly evident as principal component analysis (PCA) and hierarchical clustering revealed that the SlHa bio-replicates were much closer to Ctr and Sl, and far away from Ha replicates under 47% variance (Fig. 3A, B). Data for PCA and hierarchical clustering visualization was based on normalized counts as seen in Additional file 3.

**Table 1:** RNA sequencing and mapping results to *Pinus taeda* genome. Scots pine seedlings without fungal inoculation (Ctr), and seedlings inoculated with *H. annosum* (Ha), *S. luteus* (Sl), and both (SlHa). Each treatment condition has three biological replicates

Sample ID	Number of fragments (in million)	Number of bases (in Gb)	Number of mapped fragments (in million)	Proportion of sequenced fragments (in %)	Number of fragments used for expression quantification (in million)	Proportion of fragments used for expression quantification (in %)
Ctr-4	34.944	7.042	29.7335	85.09	10.773	36.58
Ctr-6	34.812	7.025	29.9565	86.05	10.547	35.69
Ctr-7	40.255	8.119	34.1495	84.83	12.233	36.31
Ha-1	43.887	8.853	34.2225	77.98	12.494	36.97
Ha-3	28.883	5.81	21.246	73.56	8.024	38.11
Ha-4	34.919	7.034	26.603	76.18	10.08	38.25
Sl-4	51.438	10.275	42.967	83.53	17.068	40.19
Sl-7	33.48	6.741	28.2565	84.4	10.338	36.94
Sl-10	32.366	6.518	27.1815	83.98	9.731	36.15
SlHa-2	27.209	5.476	22.631	83.18	8.253	36.77
SlHa-3	35.059	7.076	29.53	84.23	10.173	34.83
SlHa-4	54.151	10.826	44.958	83.02	17.437	39.32

**Fig. 4:**
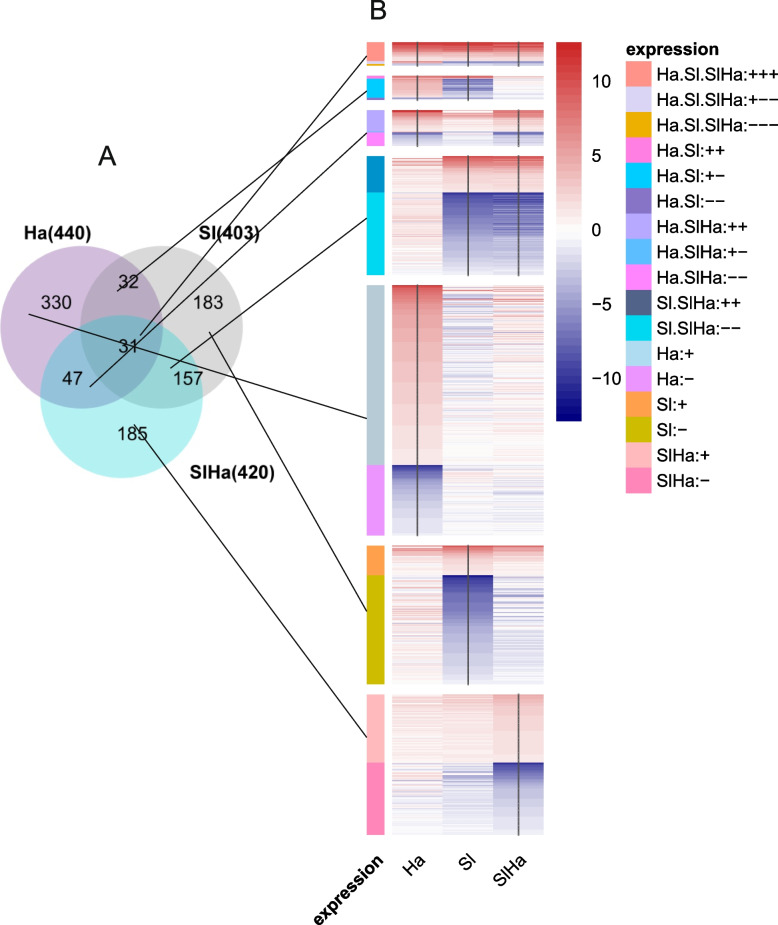
**A** 440, 403, 420 DEGs were found in Ha, Sl, SlHa relative to Ctr. 330, 183 and 185 DEGs were specific in Ha, Sl, SlHa respectively. 31 DEGs are common genes which were shared in Ha, Sl, SlHa. 32, 47, 157 DEGs were overlapped between Ha and Sl, Ha and SlHa, Sl and SlHa. **B** Hierarchical clustering of DEGs using log2foldchang based on Venn diagram. Specific genes, overlap genes between two conditions, and common genes shared in three conditions were illustrated in heatmap and each group can be divided into downregulated genes and upregulated genes. For example, 330 DEGs specifically in Ha in Venn diagram were grouped in upregulated genes and downregulated genes in the heatmap. Ctr: non-inoculated seedlings control. Ha: pathogen-infected seedlings. Sl: *Suillus luteus*-infected seedlings. SlHa: co-inoculated seedlings with both *S. luteus* and *H.annosum*. ‘ + ’ refers to upregulated, while -’ refers to downregulation. Ha.Sl.SlHa: + + + refers to DEGs that were upregulated in all treatments compared to Ctr. Ha.Sl: + + refers to DEGs that were upregulated both in Ha and in Sl. Ha.SlHa: +-refers to DEGs that were upregulated in Ha but downregulated in SlHa. Scale bar refers to log2foldchang. Red indicates a high level of upregulation, while blue indicates a high level of downregulation. Asterisks indicate whether significant difference exists in gene expression level of treatment such as Ha, Sl, SlHa compared to that of Ctr. (*p* < 0.05: *)

The expression of genes obtained by DESeq2's was shown in the heatmap (Additional file 4) using the Log2(1 + normalized CPM). As seen in the heatmap, Ha had a unique expression profile, largely different from Ctr, Sl, and SlHa**.** This validated the balancing buffer role and counteracting effect of *S. luteus*. We extracted the significant differentially expressed genes (DEGs) in Ha, Sl, SlHa comparing against Ctr with edgeR. About 440 DEGs were found in Ha, 403 in Sl, and 420 in SlHa (Fig. 4A). 31 common DEGs were found in Ha, Sl, and SlHa. Additionally, Sl shared 157 DEGs with SlHa, and most genes were downregulated (Fig. 4B). However, Ha shared only 32 DEGs with Sl and 47 DEGs with SlHa (Fig. 4A). The original data about normalized CPM, log2foldchange, and FDR value are shown in Additional file 5.

**Fig. 5:**
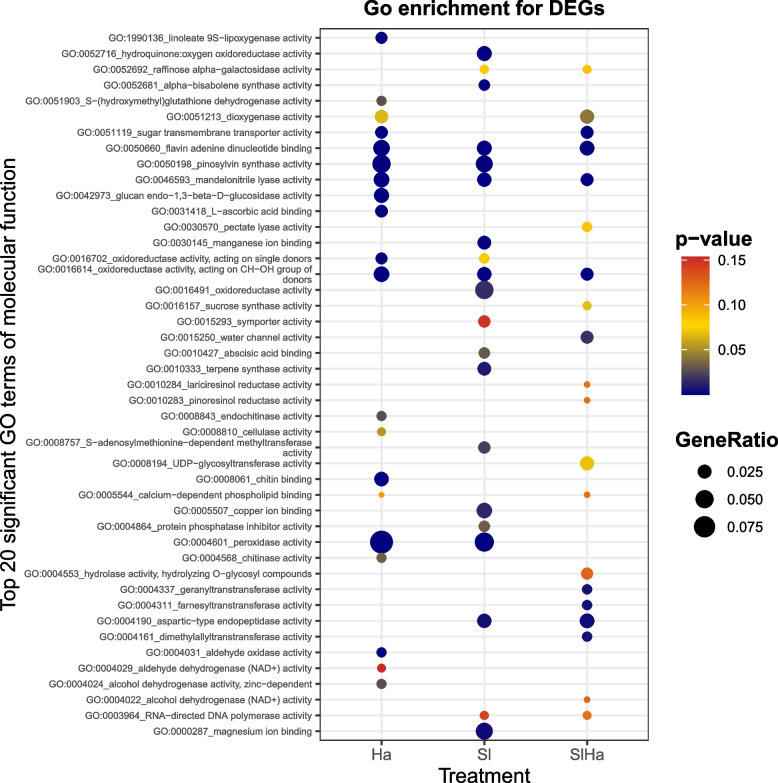
Go enrichment for DEGs–Top 20 significant GO terms of molecular function were selected in Ha, Sl, and SlHa. GeneRatio is calculated as "input gene number"/ "backgound gene number"

The best hits with annotation information were shown in Additional file 6. The top 20 significant GO terms of molecular function were selected in Ha, Sl, and SlHa (Fig. 5). The GO terms related to pinosylvin synthase activity and peroxidase activity were enriched in Ha and Sl, but not in SlHa. Many GO terms were enriched only in Ha (linoleate 9S-lipoxygenase activity, glucan endo − 1,3 − beta − D − glucosidase activity, endochitinase activity, chitin binding) or only in Sl (hydroquinone: oxygen oxidoreductase activity, manganese ion binding, magnesium ion binding, oxidoreductase activity, abscisic acid binding, terpene synthase activity, copper ion binding). The GO terms shared in the three different samples were flavin adenine dinucleotide binding, mandelonitrile lyase activity, oxidoreductase activity.

### Similarities and differences in plant response genes towards pathogenic attack and beneficial interaction

**Fig. 6:**
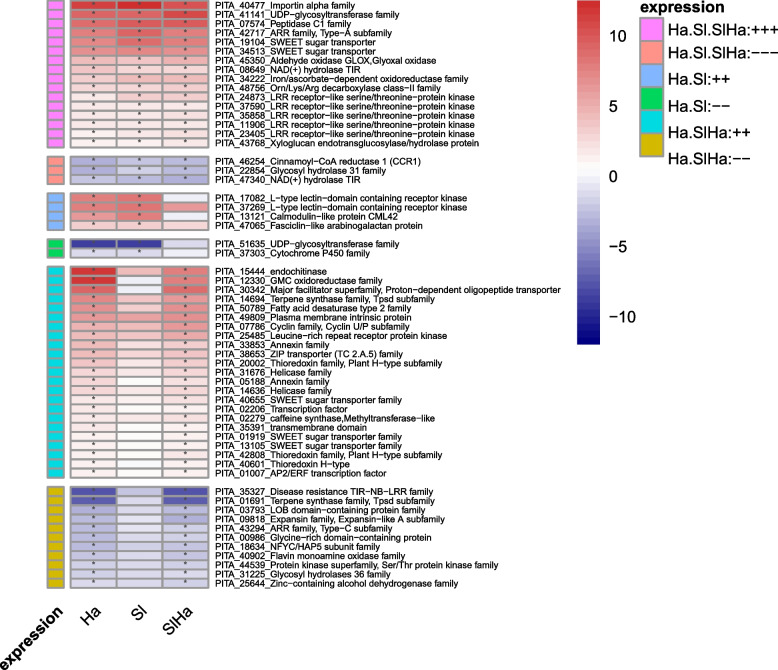
Heatmap of DEGs using log2foldchange which commonly up- or down-regulated in three treatments, and DEGs which were up- or down-regulated both in Ha and Sl, and DEGs which were up- or down-regulated in both Ha and SlHa. Scale bar refers to log2foldchang. Red indicates a high level of upregulation, while blue indicates a high level of downregulation. Asterisks indicate whether significant difference exists in gene expression level of treatment such as Ha, Sl, SlHa compared to that of Ctr. (*p* < 0.05: *)

Similar gene expression pattern was observed between Ha and Sl which was marked by the upregulation of genes related to leucine-rich repeat domain receptor-like kinases (LRR-RLKs), SWEET sugar transporters, xyloglucan endotransglucosylase/hydrolase (XTH), E3 ubiquitin ligases, UDP-glycosyltransferase (UGT), and downregulation of cinnamoyl-CoA reductase (CCR) and α-xylosidases (Fig. 6). The expression pattern of these genes was still maintained in the co-inoculation (SlHa) (Fig. 6). However, several DEGs encoding for L-type lectin-domain containing receptor kinase (LecRLKs), CML, UDP-glycosyltransferase (UGT), cytochrome P450, that were found in Ha and Sl, were not significantly expressed in co-inoculation (SlHa) (Fig. 6).

**Fig. 7:**
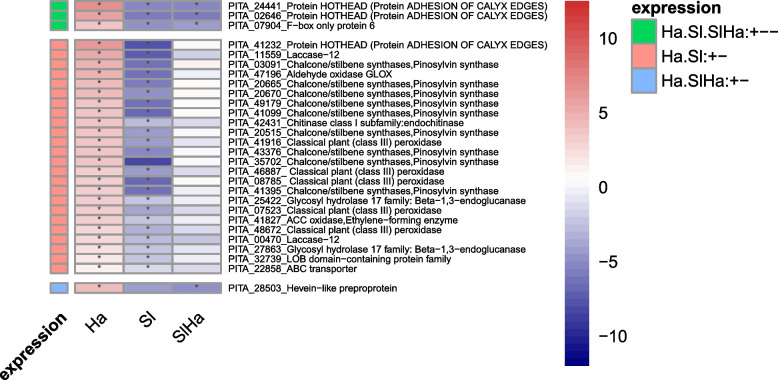
Heatmap of DEGs using log2foldchange which were upregulated in Ha but downregulated in other conditions. Scale bar refers to log2foldchang. Red indicates a high level of upregulation, while blue indicates a high level of downregulation. Asterisks indicate whether significant difference exists in gene expression level of treatment such as Ha, Sl, SlHa compared to that of Ctr. (*p* < 0.05: *)

Genes which were upregulated in Ha but downregulated in Sl included eight predicted peroxidases, nine pinosylvin synthases belonging to chalcone/stilbene synthases, three glucosyl hydrolases related to PR proteins, two laccases, ABC transporter, protein HOTHEAD, ACC oxidase (Fig. 7). *H. annosum* infection had unique expression pattern on genes encoding pathogenesis-related (PR) proteins (peroxidases, chitinases, β -1,3-glucanases, thaumatin), phenylpropanoid pathway/lignin biosynthesis (PAL, 4CL, COMT, CCoAOMT, peroxidases, laccases, cytochrome P450s), flavonoid biosynthesis, chalcone/stilbene biosynthesis, ethylene signaling pathway, JA signaling pathway, cell remodeling and growth, transporters, fungal recognition (Additional file 7, Additional file 8).

**Fig. 8:**
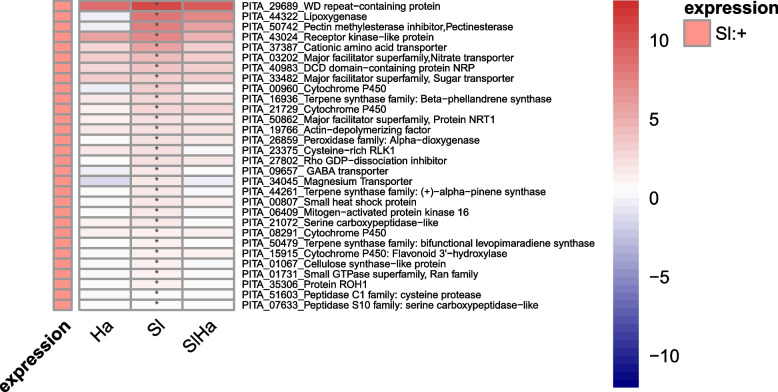
Heatmap of DEGs which were specifically upregulated in Sl. Red indicates high level of upregulation, while blue indicates high level of downregulation. Asterisks indicate whether significant difference exists in gene expression level of treatment such as Ha, Sl, SlHa compared to that of Ctr. (*p* < 0.05: *)

Whereas Sl induced a smaller unique set of genes but had more downregulated genes than Ha (Fig. 8, Additional file 9). *S. luteus* specifically induced plant genes which are mostly implicated in root growth promotion. The Sl-specific upregulated genes were associated with nutrient uptake (transporters), terpene biosynthesis and lignin biosynthesis (terpene synthases and cytochrome P450s), fungal recognition (cysteine-rich receptor-like protein kinases), hormone signaling (LOX, α-dioxygenase, MAPK) (Fig. 8). However, the Sl-specific downregulated genes were mostly encoding genes involved in plant defense responses, especially PR proteins, and other defense-related genes including laccases, chalcone/stilbene synthases, terpene synthases, cytochrome P450s, receptor-like protein kinases (RLKs) related to fungal recognition (Additional file 9). By contrast these defense related genes were not significantly expressed in SlHa co-inoculation.

### DEGs in either pathogenic attack or beneficial interaction were also maintained under co-inoculation

A set of DEGs found in Ha were also found to be maintained in SlHa (Fig. 6). Many of the upregulated genes involved in sugar transporters, annexin Gh1 related to calcium-permeable transporters, RMR function in transporting storage proteins to protein storage vacuole, ethylene-responsive transcription factors and basic endochitinase CHB4. The downregulated transcripts included genes related to glycine-rich domain-containing protein, expansin-like A, disease-resistance locus receptor-like protein kinase, GH36, flavin monoamine oxidase, and Zinc-containing alcohol dehydrogenase (Fig. 6). On the other hand, Sl shared many DEGs with SlHa (Additional file 10, Additional file 11), with major downregulated genes such as receptor-like protein kinases, glycosyl hydrolases, peroxidases, laccase, ABC transporters, calcium-binding proteins (Additional file 11).

### Unique DEGs during SlHa co-inoculation

Plants pre-inoculated with *S. luteus* and subsequently challenged with *H. annosum* were not negatively affected in terms of root growth like the plants inoculated with *S. luteus* alone. Ha invested efforts in the induction of PR proteins and stilbene synthases under pathogen attack, while SlHa gene machinery was reprogrammed towards cell wall modification (e.g. XTHs, pectinesterases, chitinase-like protein, β-mannanase, GTs, UGTs, EXORDIUM-like protein), water and nutrient uptake proteins (aquaporins, aluminum-activated malate transporter 3, bidirectional sugar transporter SWEET3b, and aluminum-activated malate transporter), fungal recognition (LRR-RLKs, LecRLKs) (Additional file 12, Additional file 13). SlHa also induced a set of genes involved in phenylpropanoid/lignin biosynthesis/flavonoid biosynthesis (CcoAMT, ANR, two bifunctional pinoresinol-lariciresinol reductases, LACs, GGPP synthases, cytosolic sulfotransferase), auxin homoeostasis (WAT1-relaterd proteins, auxin-responsive protein SAUR32), and genes involved in hormone signaling (calcium uniporter protein, E3 ubiquitin ligases with U-box domain, abscisic acid receptor, LOX, Salicylic acid 3-hydroxylase) (Additional file 12). S3H can hydrolyze salicylic acid (SA) to 2,3-DHBA, a deactivated form of SA to prevent over accumulation of SA [55]. SlHa had two S3Hs upregulated but four downregulated, while Ha induced four S3Hs. This could indicate that different hormone signaling pathways were utilized by plants in response to SIHa inoculation.

DEGs specifically upregulated in SlHa were also involved in fungal recognition such as G-type LecRLKs and LRR-RLKs, disease resistance NB-LRR. By contrast some genes related to fungal recognition were found to be downregulated, including two cysteine-rich receptor-like protein kinases, three G-type LecRLKs, and three LRR-RLKs (Additional file 13).

## Discussion

In this study, although no mantle or Hartig net was formed as a sign of mycorrhization during the short period of the experiment, the beneficial impact of *S. luteus* in promoting plant primary root growth was still evident. Other authors previously reported that *S. luteus* mycorrhiza enhanced plant growth by taking up a greater quantity of phosphorus than non-mycorrhizal roots of young seedlings of *P. radiata* [56]. To ensure successful colonization of the roots, ECM fungus *S. luteus* mycelia and plant seedlings were left to grow together for a month on artificial media before transfer into the soil. No barrage or demarcation zone was observed in the dual culture of *S. luteus* and *H. annosum*, which suggested that *S. luteus* probably had no antagonism or antibiosis effect on *H. annosum* growth. Sillo et al. (2015) [57] noted that *Heterobasidion* spp. isolates always completely overgrew *S. luteus*.

The interactions between the Scots pine seedling roots with either the pathogen or the beneficial fungus were most likely regulated by pathogen or microbe associated molecular patterns (PAMP/MAMP). Most often plants deploy plasma membrane-localized pattern recognition receptors (PRRs) and nucleotide-binding (NB)-LRR proteins to recognize MAMPs/PAMPs, or effectors, eliciting MAMP/PAMP-triggered immunity (MTI/PTI), effector-triggered immunity (ETI) [58]. Plants could differentiate symbiotic microbes from pathogens by receptor competition and inhibit MTI for symbionts [59]. LRR-RLKs and LecRLKs probably served as PRRs [58–60]. In our study, the expression of gene encoding LRR-RLKs and L-type LecRLKs in both Ha and Sl may indicate the perception of conserved MAMPs in *H. annosum* and *S. luteus*. Chitin and β-glucan are typical fungal MAMPs [61, 62]. SWEET sugar transporters were found to be expressed in Ha and Sl in this study. SWEETs facilitate sugar flux across the cell membrane [63], and some SWEETs can bind bacterial effectors [64]. The sugar efflux function of SWEET transporters probably help pathogens and symbionts for their nutrition [65]. PtaSWEET1c, which was identified in *Populus tremula* × *alba*–*Laccaria bicolor* symbiosis, was localized in the host plasma membrane surrounding the Hartig net to unload glucose and sucrose to meet the nutritional demands of colonizing hyphae [66].

Most DEGs in Ha were upregulated while most DEGs in Sl were downregulated in this study, which might suggest that beneficial fungus could elicit weaker gene expression changes compared to pathogen-induced responses [59]. The results in this study also showed that defense-related genes were suppressed by *S. luteus* but were induced by *H. annosum*, indicating that *S. luteus* promoted mutualistic interaction by suppressing plant defense responses. *H. annosum* infection induced unique gene expression patterns in PR proteins, phenylpropanoid pathway/lignin biosynthesis, flavonoid biosynthesis, chalcone/stilbene biosynthesis, while ECM fungus inoculation repressed defense-related genes (peroxidases, laccases), receptor-like protein kinases, methyltransferases, germin-like proteins (GLPs). Methyltransferases work on cytosine methylation that influences gene expression and represses transcription [67]. Plant GLPs are associated with enzymatic activities including oxalate oxidase (OxO), superoxide dismutase (SOD), and ADP glucose pyrophosphatase (AGPPase) [68], playing crucial roles in disease resistance and defense responses under biotic stress [69]. GLP2 was induced in Norway spruce trees in response to *Heterobasidion* infection [44]. Unlike in plant-pathogenic interaction wherein plant immunity can be triggered against pathogen infection, plant immunity is often suppressed in the plant-mutualistic interaction to facilitate successful colonization [70]. For example, peroxidase activity in Norway spruce roots was induced in response to pathogenic *Ceratocystis polonica* but was evaded or suppressed during interaction with the ECM fungus *L. bicolor* [71]. In *P. sylvestris*-*L. bicolor* interaction, genes involved in cell wall modification (XTH and β-xylosidase) were expressed and antifungal α-pinene synthase was downregulated [72]. The support for our observation was previously noted in *Populus* and *L.bicolor* interaction where poplar JA-responsive defense gene expressions were blocked by symbiotic effectors (MiSSP7) from *L. bicolor*. This gene prevented the JA repressor PtJAZ6 degradation during symbiosis development [73, 74]. The symbiotic effector promotes the symbiotic interaction probably through maintaining the repression of transcription factor PtMYC2.1‐regulated genes [75]. Overexpression of poplar MYC2s impaired the fungal growth and the formation of Hartig net in planta, activating the expression of defensive genes encoding terpene synthases, chitinases, GLPs, LRR-RLKs, β-glucosidase and ERF/AP2 [76]. Furthermore, additional supporting evidence to our study is found in oak-ECM fungi interaction. In this system, oak growth was enhanced by three EMC fungi *P. microcarpus*, *P. involutus* and *L. bicolor,* with a common reduction of core DEGs in colonized roots including genes encoding proteins involved in carbon metabolism, defense responses, phenolic pathways and transport [11]. Local host defenses may be activated transiently by MAMPs of ECM fungi like chitins, but plant defense genes could be repressed at the later developmental stage to benefit ECM fungal growth [30].

In this study, *S. luteus* inoculation induced genes related to nutrient transporters, such as amino acids, nitrates, sugar, and magnesium. This may indicate the fungus could promote plant growth by enhancing nutrient uptake. During poplar—*L. bicolor* interaction, ethylene and jasmonic acid pathways were induced at the late stage of root colonization to limit fungal growth within roots [77]. The induction of terpene synthases, cytochrome P450s, LOX, MPAK by *S. luteus* inoculation in our study may suggest that plant limited *S. luteus* growth in roots by terpene biosynthesis pathway and hormone signaling. Auxin signaling was activated in poplar during *L. bicolor* colonization, which could facilitate root growth [78]. In our study, defense response-related genes and cell wall modification-related genes were induced by co-inoculation, suggesting plants had balancing buffering defense responses and growth under co-inoculation. Auxin homoeostasis-related genes (WAT1-relaterd proteins, auxin-responsive protein SAUR32) were upregulated in SlHa, which may contribute to the primary root growth.

## Conclusions

*S. luteus* promoted mutualistic interaction by suppressing plant defense responses. Pre-inoculation of Scots pine seedlings with beneficial fungus *S. luteus* prior to pathogen challenge promoted primary root growth, as well as had a balancing buffering role in plant defense responses and cell growth at transcriptome level.

## Supplementary Information

Additional file 1. Dual culture of *S. luteus* and *H. annosum* on MMN agar media. *S. luteus* had grown for 13 days prior to the inoculation of *H. annosum*. Photos were taken 7 days and 17 days after *H. annosum* colonization.

Additional file 2. All seedlings that were recorded from all treatments. Information includes ID, primary root length, and lateral root number. NA refers to not available.

Additional file 3. Normalized counts that were obtained by DESeq2's median of ratios.

Additional file 4. Hierarchical clustering of DEGs using log2(1 + TMM-normalized CPM). CPM: Counts per million. CPM = raw count of each gene/lib.size of each sample. We keep genes that are expressed at least 1 CPM in at least 3 libraries in differential gene expression analysis using EdgeR. Ctr: control seedlings without any inoculum. Ha: pathogen-infected seedlings. Sl: *Suillus luteus* -infected seedlings. SlHa: co-infected seedlings with both *S. luteus* and *H.annosum* . ‘ + ’ refers to upregulated, while ‘-’ refers to downregulation. Ha.Sl.SlHa: + + + refers to DEGs that were upregulated in all treatments compared to Ctr. Ha.Sl: + + refers to DEGs that were upregulated both in Ha and in Sl. Ha.SlHa: +—refers to DEGs that were upregulated in Ha but downregulated in SlHa. The value on the scale bar refers to log2(1 + TMM-normalized CPM).

Additional file 5. Summary of DEGs into common genes in three treatments, genes overlapped in two treatments, and treatment-specific genes based on the data from the Venn diagram in Fig. 4 A. The data in each file included Gene ID, TMM-normalized CPM in replicates of each treatment, logFC, FDR, protein family, and protein name. ‘1’ refers to upregulated, while ‘0’ refers to downregulation. 31_000 refers to DEGs that were upregulated in all treatments compared to Ctr. 32_11 refers to DEGs that were upregulated in both Ha and in Sl. 330_1 refers to DEGs that were specifically upregulated in Ha.

Additional file 6. Best hit selected by bit score for each CDS sequence of DEGs which blast against to Scots pine de novo transcriptome assembly fasta sequences constructed with Trinity (Liu et al., 2022).

Additional file 7. Heatmap of DEGs which were specifically upregulated in Ha. Red indicates a high level of upregulation, while blue indicates a high level of downregulation. Asterisks indicate whether significant difference exists in gene expression level of treatment such as Ha, Sl, SlHa compared to that of Ctr. (*p* < 0.05: *).

Additional file 8. Heatmap of DEGs which were specifically downregulated in Ha. Red indicates a high level of upregulation, while blue indicates a high level of downregulation. Asterisks indicate whether significant difference exists in gene expression level of treatment such as Ha, Sl, SlHa compared to that of Ctr. (*p* < 0.05: *).

Additional file 9. Heatmap of DEGs which were specifically downregulated in Sl. Red indicates a high level of upregulation, while blue indicates high level of downregulation. Asterisks indicate whether significant difference exists in gene expression level of treatment such as Ha, Sl, SlHa compared to that of Ctr. (*p* < 0.05: *).

Additional file 10. Heatmap of DEGs using log2foldchange which were upregulated in both Sl and SlHa. Red indicates a high level of upregulation, while blue indicates a high level of downregulation. Asterisks indicate whether significant difference exists in gene expression level of treatment such as Ha, Sl, SlHa compared to that of Ctr. (*p* < 0.05: *).

Additional file 11. Heatmap of DEGs using log2foldchange which were downregulated in both Sl and SlHa. Red indicates a high level of upregulation, while blue indicates a high level of downregulation. Asterisks indicate whether significant difference exists in gene expression level of treatment such as Ha, Sl, SlHa compared to that of Ctr. (*p* < 0.05: *).

Additional file 12. Heatmap of DEGs which were specifically upregulated in SlHa. Red indicates a high level of upregulation, while blue indicates a high level of downregulation. Asterisks indicate whether significant difference exists in gene expression level of treatment such as Ha, Sl, SlHa compared to that of Ctr. (*p* < 0.05: *).

Additional file 13. Heatmap of DEGs which were specifically downregulated in SlHa. Red indicates a high level of upregulation, while blue indicates a high level of downregulation. Asterisks indicate whether significant difference exists in gene expression level of treatment such as Ha, Sl, SlHa compared to that of Ctr. (*p* < 0.05: *).

## Data Availability

The data sets generated and analyzed during this study are available in the NCBI SRA database (https://www.ncbi.nlm.nih.gov/sra) under the accession number PRJNA1143718 (https://www.ncbi.nlm.nih.gov/sra/PRJNA1143718).

## Abbreviations

ECM: Ectomycorrhizal
PR: Pathogenesis-related
JA: Jasmonic acid
SA: Salicylic acid
DEG: Differentially expressed genes
LRR-RLKs: Leucine-rich repeat domain receptor-like kinases
LecRLKs: Lectin-domain containing receptor kinase
SWEETs: Sugar will eventually be exported transporters
XTH: Xyloglucan endotransglucosylase/hydrolase
UGT: UDP-glycosyltransferase
ABC: ATP-binding cassette
SWEET: Sugars Will Eventually be Exported Transporters
