# Supplementary material for: Beneficial mutualistic fungus Suillus luteus provided excellent buffering insurance in Scots pine defense responses under pathogen challenge at transcriptome level
Source: BMC Plant Biol. 2025 Jan 3;25:12. doi: 10.1186/s12870-024-06026-z (PMC11697944; doi:10.1186/s12870-024-06026-z)
Supplement: Supplementary file 1 — Additional file 1. Dual culture of S. luteus and H. annosum on MMN agar media. S. luteus had grown for 13 days prior to the inoculation of H. annosum. Photos were taken 7 days and 17 days after H. annosum colonization. [file 12870_2024_6026_MOESM1_ESM.pdf]

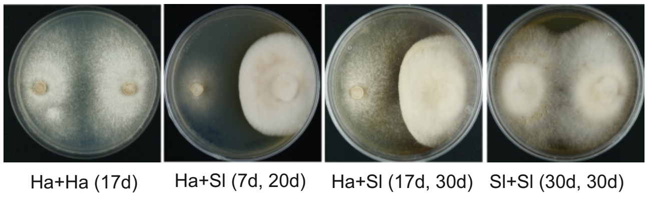

Additional file 1. Dual culture of *S. luteus* and *H. annosum* on MMN agar media. *S. luteus* had grown for 13 days prior to the inoculation of *H. annosum*. Photos were taken 7 days and 17 days after *H. annosum* colonization.
