# Supplementary figures and images for: Beneficial mutualistic fungus Suillus luteus provided excellent buffering insurance in Scots pine defense responses under pathogen challenge at transcriptome level
Source: BMC Plant Biol. 2025 Jan 3;25:12. doi: 10.1186/s12870-024-06026-z (PMC11697944; doi:10.1186/s12870-024-06026-z)

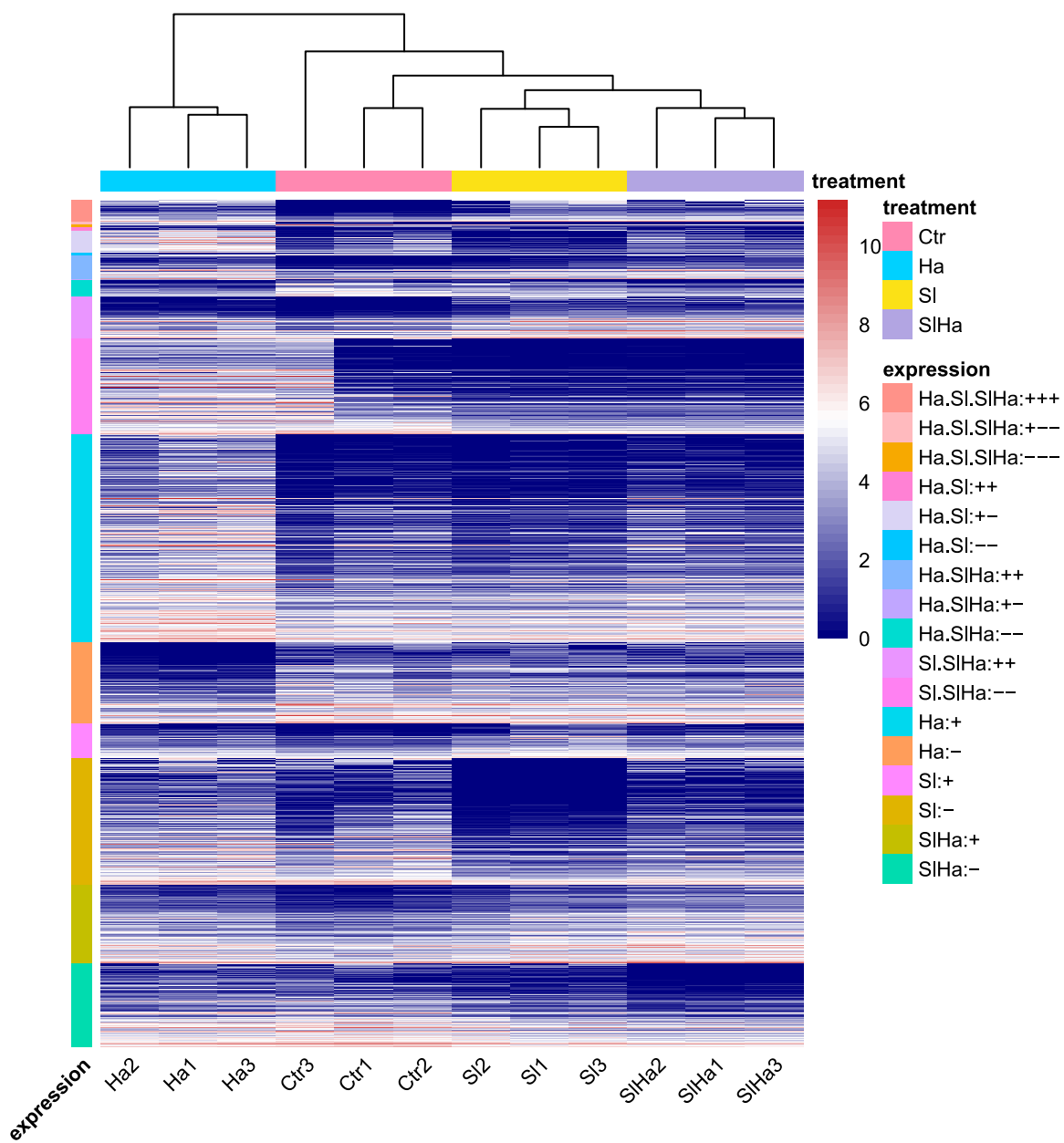

Additional file 4. Hierarchical clustering of DEGs using  $\log_2(1 + \text{TMM-normalized CPM})$ .

Supplement: Supplementary file 4 — Additional file 4. Hierarchical clustering of DEGs using log2(1 + TMM-normalized CPM). CPM: Counts per million. CPM = raw count of each gene/lib.size of each sample. We keep genes that are expressed at least 1 CPM in at least 3 libraries in differential gene expression analysis using EdgeR. Ctr: control seedlings without any inoculum. Ha: pathogen-infected seedlings. Sl: Suillus luteus -infected seedlings. SlHa: co-infected seedlings with both S. luteus and H.annosum . ‘ + ’ refers to upregulated, while ‘-’ refers to downregulation. Ha.Sl.SlHa: + + + refers to DEGs that were upregulated in all treatments compared to Ctr. Ha.Sl: + + refers to DEGs that were upregulated both in Ha and in Sl. Ha.SlHa: +—refers to DEGs that were upregulated in Ha but downregulated in SlHa. The value on the scale bar refers to log2(1 + TMM-normalized CPM). [file 12870_2024_6026_MOESM4_ESM.pdf]

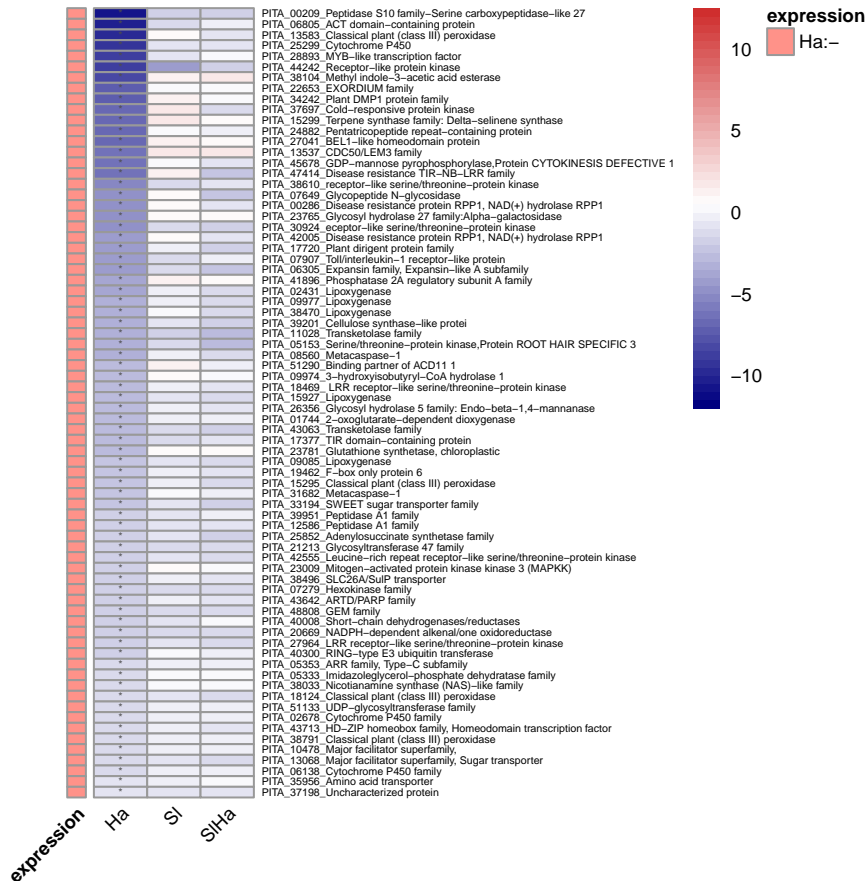

Additional file 8. Heatmap of DEGs which were specifically downregulated in Ha.

Supplement: Supplementary file 8 — Additional file 8. Heatmap of DEGs which were specifically downregulated in Ha. Red indicates a high level of upregulation, while blue indicates a high level of downregulation. Asterisks indicate whether significant difference exists in gene expression level of treatment such as Ha, Sl, SlHa compared to that of Ctr. (p < 0.05: *). [file 12870_2024_6026_MOESM8_ESM.pdf]

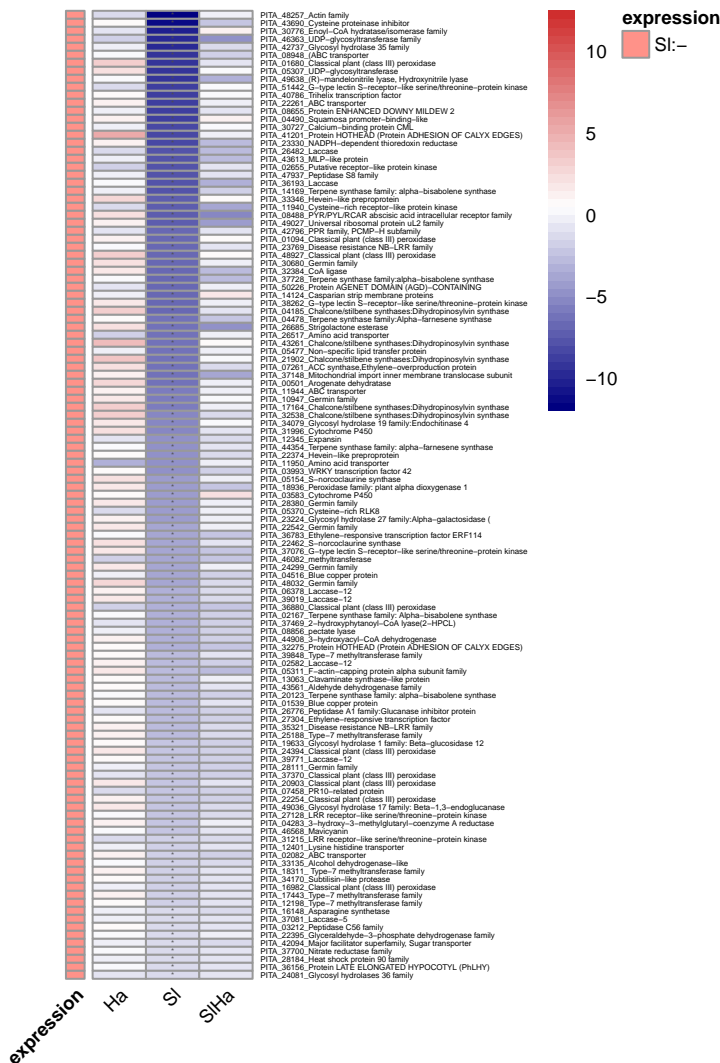

Additional file 9. Heatmap of DEGs which were specifically downregulated in SI.

Supplement: Supplementary file 9 — Additional file 9. Heatmap of DEGs which were specifically downregulated in Sl. Red indicates a high level of upregulation, while blue indicates high level of downregulation. Asterisks indicate whether significant difference exists in gene expression level of treatment such as Ha, Sl, SlHa compared to that of Ctr. (p < 0.05: *). [file 12870_2024_6026_MOESM9_ESM.pdf]

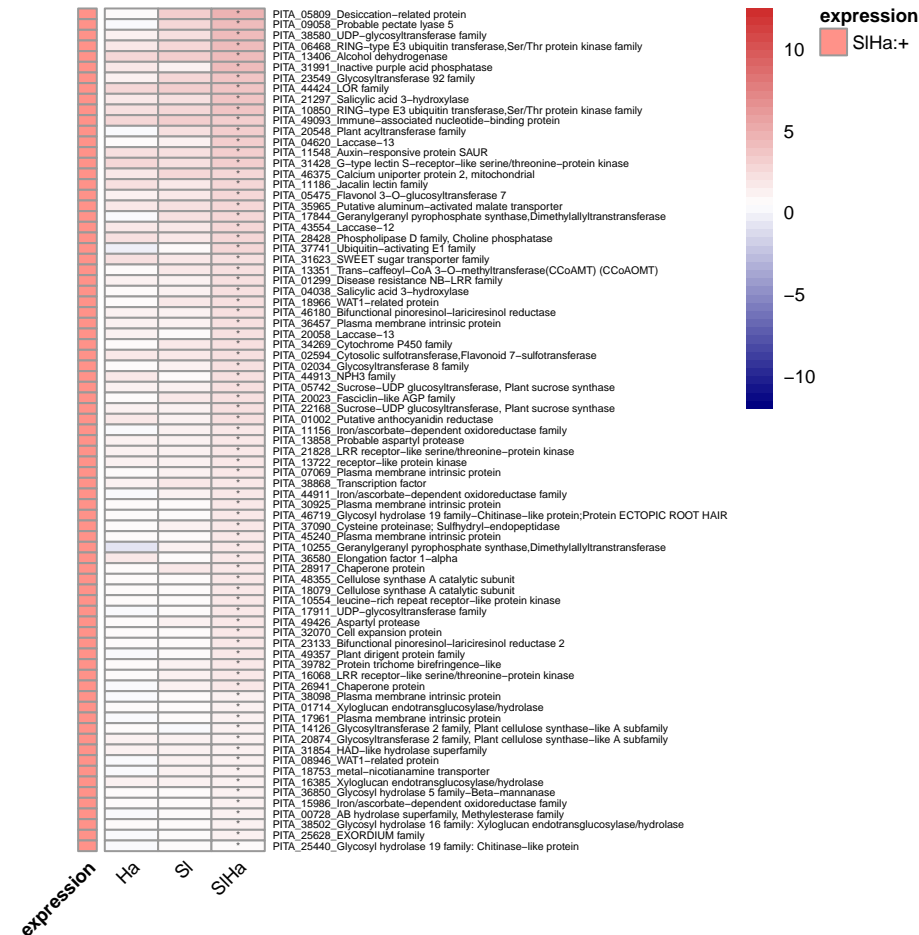

Additional file 12. Heatmap of DEGs which were specifically upregulated in SIHa.

Supplement: Supplementary file 12 — Additional file 12. Heatmap of DEGs which were specifically upregulated in SlHa. Red indicates a high level of upregulation, while blue indicates a high level of downregulation. Asterisks indicate whether significant difference exists in gene expression level of treatment such as Ha, Sl, SlHa compared to that of Ctr. (p < 0.05: *). [file 12870_2024_6026_MOESM12_ESM.pdf]

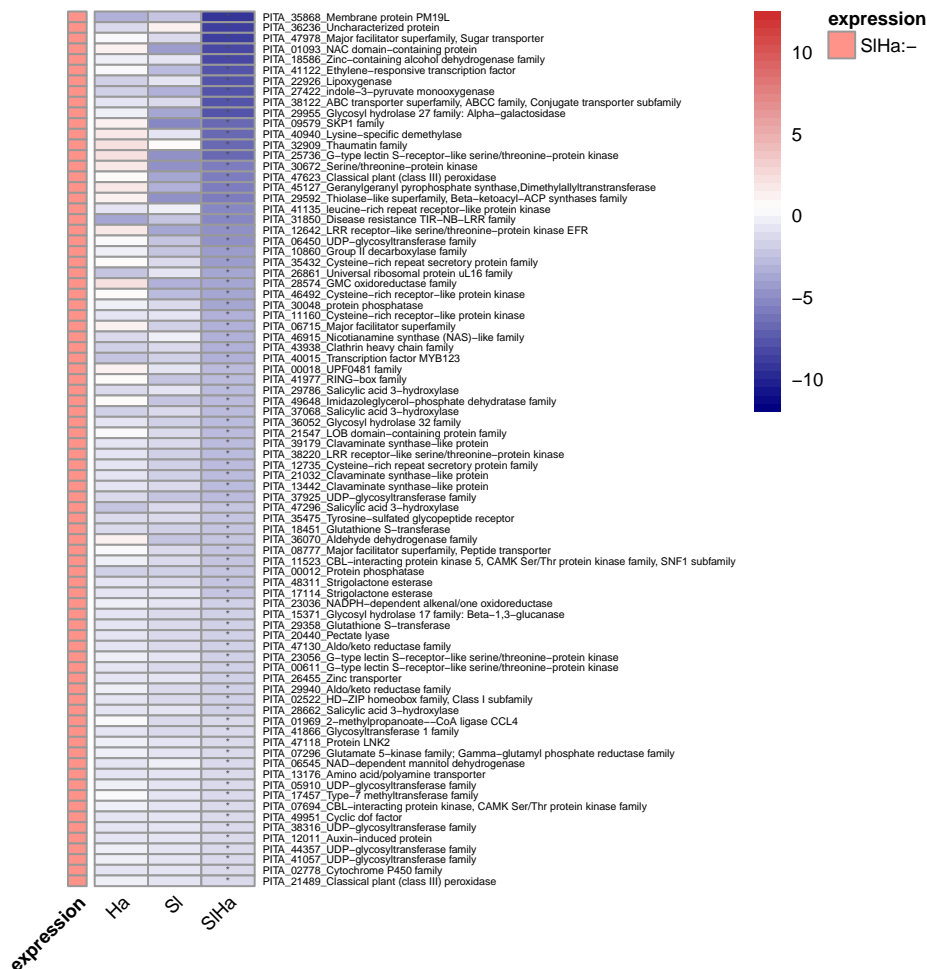

Additional file 13. Heatmap of DEGs which were specifically downregulated in SIHa.

Supplement: Supplementary file 13 — Additional file 13. Heatmap of DEGs which were specifically downregulated in SlHa. Red indicates a high level of upregulation, while blue indicates a high level of downregulation. Asterisks indicate whether significant difference exists in gene expression level of treatment such as Ha, Sl, SlHa compared to that of Ctr. (p < 0.05: *). [file 12870_2024_6026_MOESM13_ESM.pdf]
