# Supplementary material for: Beneficial mutualistic fungus Suillus luteus provided excellent buffering insurance in Scots pine defense responses under pathogen challenge at transcriptome level
Source: BMC Plant Biol. 2025 Jan 3;25:12. doi: 10.1186/s12870-024-06026-z (PMC11697944; doi:10.1186/s12870-024-06026-z)
Supplement: Supplementary file 7 — Additional file 7. Heatmap of DEGs which were specifically upregulated in Ha. Red indicates a high level of upregulation, while blue indicates a high level of downregulation. Asterisks indicate whether significant difference exists in gene expression level of treatment such as Ha, Sl, SlHa compared to that of Ctr. (p < 0.05: *). [file 12870_2024_6026_MOESM7_ESM.pdf]

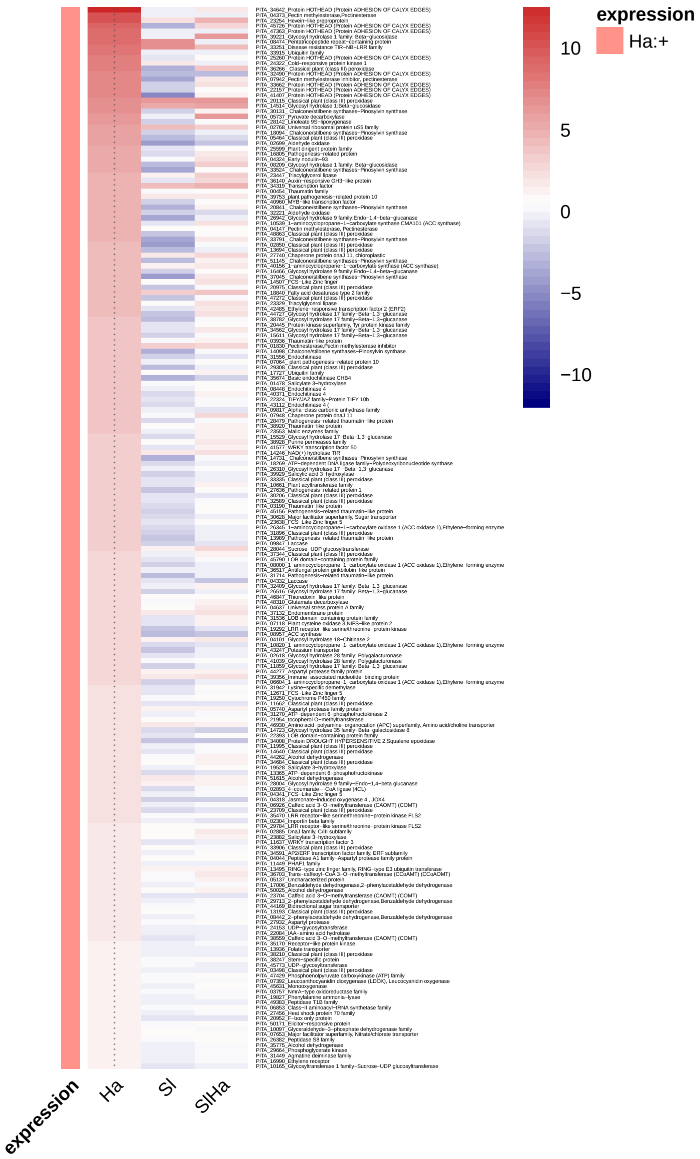

Additional file 7. Heatmap of DEGs which were specifically upregulated in Ha.
